# Supplementary material for: Prediction of adherence to treatment with statins and anti-platelet drugs in first-year post-stroke patients: Validation of beta-regression models
Source: PLoS One. 2026 Mar 26;21(3):e0345936. doi: 10.1371/journal.pone.0345936 (PMC13020832; doi:10.1371/journal.pone.0345936)
Supplement: S1 Table — (PDF) [file pone.0345936.s001.pdf]

Table S1: TEN-SPIDERS reporting tool for PDC

TEN-SPIDERS reporting tool for PDC		
**Threshold**	PDC was analyzed as a continuous variable with beta-regression	
**Eligibility criteria for inclusion in sample**	See text.	
**Numerator and denominator**	Denominator= follow up time (maximum = 365)	Numerator: Presupply + days covered after index date + hospitalization days
**Survival**	Patients who died less than 100 days of follow-up were excluded	
**Pre-supply**	90 days look back	Carried into observation period
**In-hospital supply**	Assumed	added to numerator
**Dosing information**	Available	
**Early refills**	carry-over was granted for early refills of the same drug.	overlapping days of supply were carried forward as individuals were assumed to finish any existing medication supply before commencing use of a refill of the same medication.
**Switching**	carry-over was granted for therapeutic switches (e.g. switching from simvastatin to atorvastatin).	
