# Supplementary material for: Prediction of adherence to treatment with statins and anti-platelet drugs in first-year post-stroke patients: Validation of beta-regression models
Source: PLoS One. 2026 Mar 26;21(3):e0345936. doi: 10.1371/journal.pone.0345936 (PMC13020832; doi:10.1371/journal.pone.0345936)
Supplement: S3 Table — (PDF) [file pone.0345936.s003.pdf]

Table S3: Abbreviations

Calibration In The Large	CITL
Inter Quartile Range	IQR
Mean Absolute Error	MAR
Proportion of Days Covered	PDC
Root Mean Squared Error	RMSE
Transient Ischaemic Attack	TIA
Widely Applicable Information Criterion	WAIC
