# Supplementary material for: Prediction of adherence to treatment with statins and anti-platelet drugs in first-year post-stroke patients: Validation of beta-regression models
Source: PLoS One. 2026 Mar 26;21(3):e0345936. doi: 10.1371/journal.pone.0345936 (PMC13020832; doi:10.1371/journal.pone.0345936)
Supplement: S2 Table — (PDF) [file pone.0345936.s002.pdf]

Model comparison

To assess the hypothesis that a simplified model performs as good as a full model, we performed the following analysis: For each medication group, three models were compared: 1- a model with baseline clinical, demographic and socioeconomic predictors, 2- a model with 90 days covered as single predictor, and 3- a model with baseline clinical, demographic and socioeconomic predictors and 90 days covered as predictors. The models were compared based on WAIC and Bayesian R^2. Model predictors and modelling methodology were previously described. [1] baseline clinical, demographic and socioeconomic predictors included: We thank the reviewer for this comment. T**h**e following predictors were included in the development of the models: Age, distance from primary clinic), Number of daily medications socioeconomic score, country of origin (Israel/Other), smoking status, depression diagnosis, use of any serotonin specific reuptake inhibitor, number of Flu vaccines administered in the last 5 years before index date, number of Leumit Healthcare services clinics per 10000 clients, number of pharmacies per 10000 clients.

All model comparisons were performed using 'brms' package in R. [2] Table S2 summarizes the comparison.

Table S2 Summary of model comparisons

model	WAIC	SE	Bayesian R^2	Bayesian R^2 95%crediblity interval
Statin baseline clinical, demographic and socioeconomic predictors	-7157.6	216.7	0.125	0.108-0.144
Statin 90 days covered as single predictor	-8903.6	236.7	0.646	0.634- 0.658
Statin Combined	-8926.7	235	0.655	0.642- 0.667

Antiplatelet baseline clinical, demographic and socioeconomic predictors	-4773.2	189.3	0.211	0.188-0.233
Antiplatelet 90 days covered as single predictor	-5806.8	206.5	0.541	0.524- 0.557
Antiplatelet Combined	-5888.3	204	0.561	0.545- 0.576

1. Tannous EE, Selitzky S, Vinker S, Stepensky D, Schwarzberg E. Predictive modeling of medication adherence in post myocardial infarction patients: A bayesian approach using beta-regression. *Eur J Prev Cardiol*. 2024:zwae327. https://doi.org/10.1093/eurjpc/zwae327. Accessed 10/13/2024. doi: 10.1093/eurjpc/zwae327.

2. Paul-Christian Bürkner (2017). brms: An R Package for Bayesian Multil evel Models Using Stan. Journal of Statistical Software, 80(1, 1-28. doi: https://doi.org/10.18637/jss.v080.i01
