# Supplementary material for: Prediction of adherence to treatment with statins and anti-platelet drugs in first-year post-stroke patients: Validation of beta-regression models
Source: PLoS One. 2026 Mar 26;21(3):e0345936. doi: 10.1371/journal.pone.0345936 (PMC13020832; doi:10.1371/journal.pone.0345936)
Supplement: Attachment — Submitted filename: Reviewer Report.docx [file pone.0345936.s004.docx]

**Reviewer Report**

**Manuscript title:** *Prediction of adherence to treatment with statins and anti-platelet drugs in first-year post-stroke patients: validation of beta-regression models*
**Journal:** PLOS ONE
**Manuscript ID:** PONE-D-25-37363

**General Assessment**

This is a well-written and carefully conducted study addressing an important gap in the literature: the external validation of medication adherence prediction models in post-stroke patients. The authors validate previously published beta-regression models for statin and antiplatelet adherence using prescription refill data from a large Israeli healthcare provider. The manuscript is timely, clinically relevant, and in line with PLOS ONE’s scope.

The study is commendable for its large sample size, rigorous methodology, and transparent reporting of performance metrics. Importantly, external validation of adherence models is rarely performed, making this contribution novel and valuable.

That said, the manuscript could be strengthened in several areas, particularly in contextualization, clarity of reporting, and discussion of clinical implications and generalizability.

**Major Comments**

1. **Central premise of the model and prescription cycle dependence**
   - The main novelty of this work is that adherence in the first 90 days serves as the sole predictor of 1-year adherence, eliminating the need for additional demographic or socioeconomic variables. This is an elegant and clinically appealing concept, and it likely reflects habit formation.
   - However, this premise only holds in systems with **30-day prescription cycles**, where patients have three refill opportunities in the first 90 days, allowing meaningful assessment of adherence patterns.
   - In systems where **90-day fills are standard** (e.g., North America, many European countries), the “90-day adherence” measure collapses to a near-binary indicator (one fill vs. no fill). This strips away predictive resolution, meaning the model could become substantially less useful, or even unusable, in those contexts.
   - I strongly recommend that the authors:
     - Explicitly acknowledge that the model is tied to 30-day fill patterns.
     - Discuss in both the **Introduction** and **Discussion** how this limitation affects international generalizability.
     - Suggest how the model might need to be recalibrated or adapted (e.g., shorter observation windows, refill consistency measures) for settings with 90-day fills.
2. **Predictive performance and interpretation**
   - The reported explained variance values suggest that the statin model demonstrates good predictive performance, accounting for 67% of the variance in the external dataset (R² = 0.67). In contrast, the antiplatelet model explains 56% (R² = 0.56), which represents moderate but acceptable predictive ability. These values are reasonable for clinical prediction models, though the statin model appears to perform more strongly.
   - The clinical relevance of these values should be discussed more explicitly. What level of explained variance is sufficient for a model to be considered useful in clinical or health system practice?
3. **Introduction and literature context**
   - The introduction is somewhat vague and would benefit from citation of more recent and directly relevant work in adherence prediction. Currently, the background relies heavily on general epidemiology and older references. Including more up-to-date literature will strengthen the framing.
4. **Stroke vs. TIA populations**
   - The cohort includes both ischemic stroke and TIA patients (n = 2426 screened). The relative distribution is not clearly reported. These are distinct conditions, and adherence behaviors may differ.
   - I recommend either reporting separate numbers and characteristics for ischemic stroke vs. TIA patients, or ideally performing subgroup analyses. If this is not feasible, the rationale for pooling the two diagnoses should be provided.
5. **Generalizability**
   - While this is an external validation study, the dataset is drawn from a single provider (LHCS). The authors should clarify the **coverage of LHCS** (what proportion of the Israeli population it serves) and discuss implications for generalizability.
   - Moreover, **more than 94% of patients in this study had 1-month prescription cycles**. This prescribing pattern may not reflect practice in other countries, where 90-day fills are frequently the norm. This limitation should be emphasized, as it directly affects the transportability of the model internationally.
   - Ideally, validation in an additional dataset (from another provider or another country) would strengthen confidence in generalizability. At minimum, the limitations should be more explicitly acknowledged.
6. **Calibration and error metrics**
   - Calibration slopes (1.06 for statins, 0.96 for antiplatelets) are close to ideal, which is encouraging.
   - However, absolute error remains relatively high: for the antiplatelet model, 70% of predictions had an absolute error of less than 20.9%, and MAE values were 18–20%. This raises concerns about utility at the individual patient level. The authors should discuss whether the model is more appropriate for **population-level predictions** rather than individual risk stratification.
7. **Definition and measurement of adherence (PDC)**
   - PDC is an accepted metric, but its limitations need clearer emphasis. In particular:
     - Low-dose aspirin is available over the counter in Israel, which may have underestimated antiplatelet adherence.
     - Prescription fills do not guarantee actual consumption.
8. **Implications for practice**
   - The discussion suggests embedding the model in electronic health records, which is a valuable point. The authors should elaborate on *how* such an approach could be operationalized (e.g., automated alerts for case managers, linkage to adherence interventions) and whether supporting evidence exists for these strategies.

**Minor Comments**

1. **Typos and capitalization errors**:
   - Line 206: “Moreover, We showed…” → *we*
   - Line 210: “(Age, distance…)” → *age*
   - Line 213: “country of origin (Israel/Other)” → *other*
     Please proofread for similar inconsistencies.
2. **Table 1 formatting**: Variables should be specified as n (%) or median (IQR), in line with reporting standards.
3. **Terminology**: When first mentioned, “explained variance” should be explicitly linked to R² for clarity to non-technical readers.
4. **Figures**: Calibration plots are appropriate but could be improved with larger fonts and clearer legends.
5. **Data availability**: Please ensure compliance with PLOS ONE’s open data policy, or provide a stronger justification if restrictions apply.

**Recommendation**

This is a well-executed and clinically relevant validation study. With revisions to strengthen the introduction, clarify stroke/TIA subgroups, refine reporting, and—most importantly—address the central limitation of prescription cycle dependence (30-day vs. 90-day fills), the manuscript will make a strong contribution to the literature.

**Recommendation: Minor revisions**
