# Supplementary material for: Prediction of adherence to treatment with statins and anti-platelet drugs in first-year post-stroke patients: Validation of beta-regression models
Source: PLoS One. 2026 Mar 26;21(3):e0345936. doi: 10.1371/journal.pone.0345936 (PMC13020832; doi:10.1371/journal.pone.0345936)
Supplement: Attachment — Submitted filename: Reviewer Report ET 29102025_DS.docx [file pone.0345936.s005.docx]

**Reviewer Report**

**Manuscript title:** *Prediction of adherence to treatment with statins and anti-platelet drugs in first-year post-stroke patients: validation of beta-regression models*
**Journal:** PLOS ONE
**Manuscript ID:** PONE-D-25-37363

**General Assessment**

This is a well-written and carefully conducted study addressing an important gap in the literature: the external validation of medication adherence prediction models in post-stroke patients. The authors validate previously published beta-regression models for statin and antiplatelet adherence using prescription refill data from a large Israeli healthcare provider. The manuscript is timely, clinically relevant, and in line with PLOS ONE’s scope.

The study is commendable for its large sample size, rigorous methodology, and transparent reporting of performance metrics. Importantly, external validation of adherence models is rarely performed, making this contribution novel and valuable.

That said, the manuscript could be strengthened in several areas, particularly in contextualization, clarity of reporting, and discussion of clinical implications and generalizability.

**Major Comments**

1. **Central premise of the model and prescription cycle dependence**
   - The main novelty of this work is that adherence in the first 90 days serves as the sole predictor of 1-year adherence, eliminating the need for additional demographic or socioeconomic variables. This is an elegant and clinically appealing concept, and it likely reflects habit formation.
   - However, this premise only holds in systems with **30-day prescription cycles**, where patients have three refill opportunities in the first 90 days, allowing meaningful assessment of adherence patterns.
   - In systems where **90-day fills are standard** (e.g., North America, many European countries), the “90-day adherence” measure collapses to a near-binary indicator (one fill vs. no fill). This strips away predictive resolution, meaning the model could become substantially less useful, or even unusable, in those contexts.
   - I strongly recommend that the authors:
     - Explicitly acknowledge that the model is tied to 30-day fill patterns.
     - Discuss in both the **Introduction** and **Discussion** how this limitation affects international generalizability.
     - Suggest how the model might need to be recalibrated or adapted (e.g., shorter observation windows, refill consistency measures) for settings with 90-day fills.
     - We thank the reviewer for this comment. The current model is based on 30-day fill patterns. We added a paragraph to the Introduction and Discussion explaining how this limitation affects international applicability. Additionally, we suggested how to modify the model for settings with 90-day fills. We appreciate the reviewer’s point about differences in dispensing intervals across different systems. In health systems where 90-day prescriptions are standard, the first 90 days may only involve a single fill, limiting variability in early adherence. To improve the model’s relevance and comparability, we propose extending the early adherence window to 180 days, covering two refill cycles in 90-day dispensing systems. This provides a more stable measure of sustained medication use. Although this slightly delays prediction timing, it enhances robustness and cross-system applicability without altering the core concept of early adherence that underpins the model.
2. **Predictive performance and interpretation**
   - The reported explained variance values suggest that the statin model demonstrates good predictive performance, accounting for 67% of the variance in the external dataset (R² = 0.67). In contrast, the antiplatelet model explains 56% (R² = 0.56), which represents moderate but acceptable predictive ability. These values are reasonable for clinical prediction models, though the statin model appears to perform more strongly.
   - The clinical relevance of these values should be discussed more explicitly. What level of explained variance is sufficient for a model to be considered useful in clinical or health system practice?

We thank the reviewer for this comment. While we cannot specify a level of explained variance that is sufficient for model usefulness, R² = 0.67 or even R² = 0.56 in an external validation study indicate that early adherence behavior captures a large portion of the predictable variation in 1-year medication adherence, and these values are relatively high. Clinical usefulness should be evaluated through decision-analytic assessment, such as decision curve analysis. We expanded on this topic in the discussion section.

3. **Introduction and literature context**
   - The introduction is somewhat vague and would benefit from citation of more recent and directly relevant work in adherence prediction. Currently, the background relies heavily on general epidemiology and older references. Including more up-to-date literature will strengthen the framing.

We appreciate the reviewer’s comment. We revised the Introduction section as recommended.

4. **Stroke vs. TIA populations**
   - The cohort includes both ischemic stroke and TIA patients (n = 2426 screened). The relative distribution is not clearly reported. These are distinct conditions, and adherence behaviors may differ.
   - I recommend either reporting separate numbers and characteristics for ischemic stroke vs. TIA patients, or ideally performing subgroup analyses. If this is not feasible, the rationale for pooling the two diagnoses should be provided.

We appreciate the reviewer's comment. Although we recognize that these are different conditions and adherence behaviors may vary, our model links a measure of habit formation (covering the first 90 days) to 1-year PDC for a specific patient. It is expected to perform well across different indications. The original model was developed on a post-Myocardial Infarction population, and we tested it on a different condition because we assumed that the relationship between habit formation and adherence is predictive regardless of clinical condition. Additionally, pooling these two indications is justified because long-term anti-platelet and statin therapies are the same, enhancing the model's clinical usefulness. While stroke and TIA are distinct clinical presentations, they share common vascular pathology and are managed with identical secondary prevention strategies, including statins. Major guidelines (AHA/ASA, ESO, NICE) do not differentiate between them for lipid-lowering treatment, so factors affecting adherence are likely similar. Combining stroke and TIA patients improves model stability, broadens clinical applicability, and mirrors real-world practice where adherence interventions target all cerebrovascular disease patients. We have added this explanation to the methods section.

5. **Generalizability**
   - While this is an external validation study, the dataset is drawn from a single provider (LHCS). The authors should clarify the **coverage of LHCS** (what proportion of the Israeli population it serves) and discuss implications for generalizability.

We thank the reviewer for this comment. LHCS is the smallest Israeli health provider, offering health services and insurance to approximately 7.6% of the Israeli population. However, LHCS membership is representative of the Israeli population. We added this information to the methods section and also discussed its implications for generalizability.

- Moreover, **more than 94% of patients in this study had 1-month prescription cycles**. This prescribing pattern may not reflect practice in other countries, where 90-day fills are frequently the norm. This limitation should be emphasized, as it directly affects the transportability of the model internationally.

We thank the reviewer for this comment. We addressed this limitation on comment #1 and added this to the limitations section.

- Ideally, validation in an additional dataset (from another provider or another country) would strengthen confidence in generalizability. At minimum, the limitations should be more explicitly acknowledged.

We thank the reviewer for this comment. We agree that validation from another provider or from another country, and ideally by a different research group, would be necessary to improve generalizability. We added this limitation to the limitations section.

6. **Calibration and error metrics**
   - Calibration slopes (1.06 for statins, 0.96 for antiplatelets) are close to ideal, which is encouraging.
   - However, absolute error remains relatively high: for the antiplatelet model, 70% of predictions had an absolute error of less than 20.9%, and MAE values were 18–20%. This raises concerns about utility at the individual patient level. The authors should discuss whether the model is more appropriate for **population-level predictions** rather than individual risk stratification.

We thank the reviewer for this comment. We acknowledge that the absolute error is somewhat high. However, since absolute error or root mean squared error may not be ideal for outcomes that are proportions (i.e., between 0 and 1), we compared the model’s mean absolute error (MAE) with that of a null model predicting the mean outcome for all observations. This “relative MAE reduction” is similar to the concept of mean absolute scaled error (Hyndman & Koehler, 2006) and offers an interpretable measure of predictive usefulness. The statin model reduced the MAE by 51.3% compared to this baseline, indicating a significant predictive improvement. On average, the model halves the prediction error compared to simply predicting the mean proportion. For aspirin, the relative MAE reduction was 42.1%, meaning that the typical prediction error is 42% smaller than that of a model which just predicts the mean outcome for everyone. We added this to the results and discussion sections.

Hyndman, R. J., & Koehler, A. B. (2006). Another look at measures of forecast accuracy. International Journal of Forecasting, 22(4), 679–688.

7. **Definition and measurement of adherence (PDC)**
   - PDC is an accepted metric, but its limitations need clearer emphasis. In particular:
     - Low-dose aspirin is available over the counter in Israel, which may have underestimated antiplatelet adherence.
     - Prescription fills do not guarantee actual consumption.

We appreciate the reviewer's comment. We address these limitations of PDC in the limitations section.

8. **Implications for practice**
   - The discussion suggests embedding the model in electronic health records, which is a valuable point. The authors should elaborate on *how* such an approach could be operationalized (e.g., automated alerts for case managers, linkage to adherence interventions) and whether supporting evidence exists for these strategies.

We thank the reviewer for this comment. We include a discussion on how such a prediction model can be integrated into electronic health records and connected to mobile health (mHealth) interventions like phone calls, SMS text messaging, and tele-health. There is evidence that these interventions are effective in increasing adherence, and we have added this to the discussion section.

**Minor Comments**

1. **Typos and capitalization errors**:
   - Line 206: “Moreover, We showed…” → *we*
   - Line 210: “(Age, distance…)” → *age*
   - Line 213: “country of origin (Israel/Other)” → *other*
     Please proofread for similar inconsistencies.

We thank the reviewer for these comments. We have corrected the typos.

2. **Table 1 formatting**: Variables should be specified as n (%) or median (IQR), in line with reporting standards.

We thank the reviewer for this comment. We have corrected the formatting of Table 1.

3. **Terminology**: When first mentioned, “explained variance” should be explicitly linked to R² for clarity to non-technical readers.

We thank the reviewer for this comment. We amended the text to include explicit link to R².

4. **Figures**: Calibration plots are appropriate but could be improved with larger fonts and clearer legends. We thank the reviewer for this comment. We improved the font and legends.
5. **Data availability**: Please ensure compliance with PLOS ONE’s open data policy, or provide a stronger justification if restrictions apply.

We thank the reviewer for this comment. We have ensured compliance with PLOS ONE’s open data policy. The data are available upon request to the corresponding author and with permission from LHCS.

**Recommendation**

This is a well-executed and clinically relevant validation study. With revisions to strengthen the introduction, clarify stroke/TIA subgroups, refine reporting, and—most importantly—address the central limitation of prescription cycle dependence (30-day vs. 90-day fills), the manuscript will make a strong contribution to the literature.

**Recommendation: Minor revisions**
