# Supplementary material for: Prediction of adherence to treatment with statins and anti-platelet drugs in first-year post-stroke patients: Validation of beta-regression models
Source: PLoS One. 2026 Mar 26;21(3):e0345936. doi: 10.1371/journal.pone.0345936 (PMC13020832; doi:10.1371/journal.pone.0345936)
Supplement: Attachment — Submitted filename: Reviewer Report 2.docx [file pone.0345936.s006.docx]

**Reviewer Response**

I thank the authors for their detailed and thoughtful responses to my comments, as well as for the extensive revisions made to the manuscript. I have carefully reviewed the updated text, figures, and the point-by-point rebuttal.

Overall, the authors have addressed my major and minor comments thoroughly and satisfactorily.

1. **Prescription-cycle dependence and international generalizability**
   The authors have now clearly acknowledged the dependence on 30-day fill patterns and have added appropriate explanation in both the Introduction and Discussion. The proposed extension to a 180-day observation window for systems with 90-day fills is sensible and enhances clarity regarding transportability. This substantially strengthens the manuscript.
2. **Predictive performance interpretation**
   The expanded discussion of explained variance (R²), clinical usefulness, and the value of incorporating decision-analytic perspectives (e.g., decision curve analysis) improves transparency and interpretability. The clarification around the relative MAE reduction is particularly helpful.
3. **Introduction and literature context**
   The Introduction has been updated.
4. **Stroke vs. TIA populations**
   The rationale for analyzing stroke and TIA patients together is now justified.
5. **Generalizability and LHCS dataset**
   The revised Methods now clearly indicate the coverage of LHCS (7.6% of the Israeli population) and justify representativeness. The authors appropriately highlight that further validation in independent datasets and other countries is warranted.
6. **Calibration and error metrics**
   The authors appropriately contextualized the magnitude of prediction error and clarified the difference between absolute error and relative MAE reduction. The added discussion improves the reader’s ability to judge potential applications at the individual vs. population level.
7. **PDC limitations**
   The manuscript now clearly discusses the inherent limitations of PDC, including aspirin availability over the counter and the fact that dispensation does not guarantee ingestion.
8. **Implications for practice**
   The expanded section on implementation within electronic health records—including automated alerts and linkage to mHealth adherence interventions—is clear, practical, and referenced.
9. **Minor issues**
   Typographical errors, table formatting, terminology clarification, and figure readability have been addressed.

**Overall Recommendation**
The manuscript has been substantially improved.
