# Supplementary material for: Prediction of adherence to treatment with statins and anti-platelet drugs in first-year post-stroke patients: Validation of beta-regression models
Source: PLoS One. 2026 Mar 26;21(3):e0345936. doi: 10.1371/journal.pone.0345936 (PMC13020832; doi:10.1371/journal.pone.0345936)
Supplement: Attachment — Submitted filename: response second review revised.docx [file pone.0345936.s007.docx]

We thank the reviewers for their insightful and helpful comments.

Response for Reviewer #1:

We thank reviewer #1 for the thorough and thoughtful review.

Response for Reviewer #2:

Abstract concludes that the model “may be used for adherence prediction,” which is accurate but vague. Given that this is an external validation study, readers expect at least a brief indication of how the model would be operationalized in practice (e.g., early identification of high-risk patients within 90 days post-stroke).

We appreciate the reviewer for this valuable recommendation. The abstract has been revised to clearly state that the model can be used for early detection of patients at increased risk of non-adherence within the first 90 days after a stroke, helping to enable prompt and targeted adherence-support interventions.

Introduction relies heavily on the authors’ prior myocardial infarction study as the conceptual backbone of the current work. While this is understandable given the study design, it subtly shifts the narrative from hypothesis-driven inquiry to confirmatory extension.

We appreciate this observation. As the reviewer pointed out, since the study serves as an external validation of previously developed models, it relies heavily on our earlier published work. However, this is not the only reason. We conducted an extensive literature review and found very few studies focusing on predictive modeling of medication adherence in post-TIA/stroke patients. Additionally, no other studies utilizing beta-regression for adherence prediction were identified. These are the primary reasons we heavily relied on our previous study in the introduction.

There are subtle methodological choices that deserve more explicit discussion. The overwhelming predominance of 1-month refill patterns simplifies modeling but also constrains generalizability. While this is acknowledged later as a limitation, the methods section could benefit from explicitly stating that the model’s structure is intrinsically linked to refill cadence. The absence of identified bleeding events in the antiplatelet cohort is another point that requires clarification.

We agree that these methodological features need a clearer description in the Methods section. We revised the methods to explicitly state that the model structure is directly connected to the monthly refill schedule, reflecting the dominant pattern of 1-month dispensing in the dataset .

Regarding bleeding events, we clarified in the results section that no bleeding events were identified based on available ICD-9 documentation. This may reflect limited sensitivity rather than a true absence. We also acknowledged that minor bleeding events could have occurred but were not serious enough for patients to seek medical advice.

Results section largely reports metrics without interrogating their implications. High R² values are expected when early adherence is used to predict later adherence, yet this inherent predictability is not explicitly acknowledged.

We thank the reviewer for this comment. We expanded on the implications of the metrics in the results section. We appreciate the reviewer’s comment and agree that high R² values are expected when early adherence is used to predict later adherence. We added an explicit acknowledgment of this fact to the results section. We also cited previous studies showing that adding early adherence to prediction models improves model performance. However, in the present study, which is an external validation study, a high R² value is not trivial since we did not fit a new model to the data but tested a previously developed model. Thus, a high R² in the external validation suggests that the mean relation between early adherence and 1-year adherence is relatively stable across patient populations. Typically, even well-performing models with high R² in the development dataset show significant reductions in R² when validated externally. Therefore, the high R² in the external validation dataset indicates a stable predictor-outcome relationship across populations and demonstrates that the model is transportable. We added a summary of this discussion to the results section.
