# Supplementary material for: Prediction of adherence to treatment with statins and anti-platelet drugs in first-year post-stroke patients: Validation of beta-regression models
Source: PLoS One. 2026 Mar 26;21(3):e0345936. doi: 10.1371/journal.pone.0345936 (PMC13020832; doi:10.1371/journal.pone.0345936)
Supplement: Attachment — Submitted filename: reponse to reviewrs 3.docx [file pone.0345936.s008.docx]

We thank the reviewer for these valuable comments and suggestions.

1. Line 288: these patients with 2 and 3-month days supply were still included in the validation then?

Yes, these patients were included. Since there were relatively very few patients who had a 2 or 3-month supply, we highlighted the fact that these models are more suitable for a 1-month supply. But in the validation cohort we included the (few) patients with 2 and 3-month.
2. Line 299: can you put the socioeconomic status in context, ie. were most patients middle-class then?

We thank the reviewer for this comment. Yes, most patients could be considered middle class. We added this to the results section.
3. Line 301: can you provide any explanation for why the PDC is so low? In the limitations you note that antiplatelets may be underestimated due to over-the-counter meds, but this is the statin cohort. Also why does PDC get better over time, wouldn't you expect it to be highest in the first 90 days and then trail off? Also by "new users" you mean these patients were not on statins prior to experiencing stroke? Related, why do you think PDC was so much higher for the antiplatelet cohort?

We thank the reviewer for these comments.

can you provide any explanation for why the PDC is so low?

Unfortunately, we didn`t find studies that dealt with the question of the underlying reasons of non-adherence or lower adherence to statins post TIA/stroke. In contrast, in post-myocardial infarction patients, the median was higher (PDC of 0.8). (1) We can only speculate that this may be related to how patients perceive the risk of recurrence in these two different indications.

1-Tannous EE, Selitzky S, Vinker S, Stepensky D, Schwarzberg E. Predictive modelling of medication adherence in post-myocardial infarction patients: a Bayesian approach using beta-regression. *Eur J Prev Cardiol*. 2025;32(8):649-658. doi:10.1093/eurjpc/zwae327

Also why does PDC get better over time, wouldn't you expect it to be highest in the first 90 days and then trail off?

The difference is between the median PDC at 90 days and the median PDC at 1 year, primarily in the antiplatelet cohort. The main reason is that in the antiplatelet group, some patients started filling aspirin several months after the event. While the expected pattern is high PDC initially then tapering off, the adherence literature shows a group of patients characterized by early decline and partial recovery. (2)

2-Salmasi S, De Vera MA, Safari A, et al. Longitudinal Oral Anticoagulant Adherence Trajectories in Patients With Atrial Fibrillation. *J Am Coll Cardiol*. 2021;78(24):2395-2404. doi:10.1016/j.jacc.2021.09.1370

? Also by "new users" you mean these patients were not on statins prior to experiencing stroke?

Yes, “new users” are patients who were not on statins prior to experiencing stroke as opposed to “prevalent users”.

Related, why do you think PDC was so much higher for the antiplatelet cohort?

Here, we can only speculate about the reason. We think one possibility is that patients perceive the underlying cause of the stroke/TIA as related to blood clots, and therefore believe that antiplatelets are more important than statins in this context.

4. Line 435: In what country, can you put these results in context?

We appreciate the reviewer's comment. The referenced study used data from Australia. We have added this clarification to the manuscript.
5. Line 443: typo should be medication not mediation

We thank the reviewer for this comment. We corrected the typo.

6. Line 487: this isn't really supported by the results of the current study looking at external validation, rather the baseline variables being poor predictors of adherence was reported in a prior study.

We thank the reviewer for this comment. We agree and have revised the sentence to “the present study demonstrates that 1-year adherence can be reliably predicted from data on the first 90 days of adherence”.
7. Figures are somewhat unclear and should be higher quality

We thank the reviewer for this comment. We increased the dpi from 300 to 600.
